# Low CSF Levels of Both α-Synuclein and the α-Synuclein Cleaving Enzyme Neurosin in Patients with Synucleinopathy

**DOI:** 10.1371/journal.pone.0053250

**Published:** 2013-01-08

**Authors:** Malin Wennström, Yulia Surova, Sara Hall, Christer Nilsson, Lennart Minthon, Fredrik Boström, Oskar Hansson, Henrietta M. Nielsen

**Affiliations:** 1 Lund University, Department of Clinical Sciences Malmö, Molecular Memory Research Unit, The Wallenberg Laboratory at Skåne University Hospital, Malmö, Sweden; 2 Lund University, Department of Clinical Sciences Lund, Neurology Clinic at Skåne University Hospital, Malmö, Sweden; 3 Lund University, Department of Clinical Sciences Malmö, Clinical Memory Research Unit, Memory Clinic at Skåne University Hospital, Malmö, Sweden; 4 Mayo Clinic College of Medicine, Department of Neuroscience, Jacksonville, Florida, United States of America; Nathan Kline Institute and New York University School of Medicine, United States of America

## Abstract

Neurosin is a protease that *in vitro* degrades α-synuclein, the main constituent of Lewy bodies found in brains of patients with synucleinopathy including Parkinson's disease (PD) and dementia with Lewy bodies (DLB). Several studies have reported reduced cerebrospinal fluid (CSF) levels of α-synuclein in synucleinopathy patients and recent data also proposes a significant role of α-synuclein in the pathophysiology of Alzheimer's disease (AD). To investigate potential links between neurosin and its substrate α-synuclein *in vivo* we used a commercially available sandwich ELISA and an in-house developed direct ELISA to quantify CSF levels of α-synuclein and neurosin in patients diagnosed with DLB, PD and PD dementia (PDD) versus AD patients and non-demented controls. We found that patients with synucleinopathy displayed lower CSF levels of neurosin and α-synuclein compared to controls and AD patients. In contrast, AD patients demonstrated significantly increased CSF α-synuclein but similar neurosin levels compared to non-demented controls. Further, CSF neurosin and α-synuclein concentrations were positively associated in controls, PD and PDD patients and both proteins were highly correlated to CSF levels of phosphorylated tau in all investigated groups. We observed no effect of gender or presence of the apolipoprotein Eε4 allele on neither neurosin or α-synuclein CSF levels. In concordance with the current literature our study demonstrates decreased CSF levels of α-synuclein in synucleinopathy patients versus AD patients and controls. Importantly, decreased α-synuclein levels in patients with synucleinopathy appear linked to low levels of the α-synuclein cleaving enzyme neurosin. In contrast, elevated levels of α-synuclein in AD patients were not related to any altered CSF neurosin levels. Thus, altered CSF levels of α-synuclein and neurosin in patients with synucleinopathy versus AD may not only mirror disease-specific neuropathological mechanisms but may also serve as fit candidates for future biomarker studies aiming at identifying specific markers of synucleinopathy.

## Introduction

The neuronal protein α-synuclein has been the focus of interest in several recent studies on patients with neurodegenerative disorders [1]. The protein is a member of the synuclein family and has been identified as the major component in Lewy bodies and Lewy neurites [2], [3], both neuropathological hallmarks of patients with Parkinsons's disease (PD) and dementia with Lewy bodies (DLB) [4], [5]. Altered α-synuclein synthesis, seen as cytoplasmic inclusions in glial cells, has also been found in patients with multiple system atrophy (MSA) [6], [7], [8] and collectively these disorders are referred to as synucleinopathies [9]. The α-synuclein protein is detectable in cerebrospinal fluid (CSF) and has been suggested as a potential biomarker candidate of synucleinopathy. We and others have shown that CSF concentrations of α-synuclein appear lower in patients with synucleinopathy versus controls and AD patients [10], [11], [12], [13], [14], [15], [16]. In contrast, CSF α-synuclein levels were recently shown to be significantly elevated in AD patients [14]. In support, recent evidence proposed soluble α-synuclein as a modulator of AD pathophysiology where amyloid-β and tau in synergy may foster increased α-synuclein levels leading to impaired neurotransmitter release and subsequent cognitive decline [17]. The same study also showed that the extracellular levels of α-synuclein in post mortem human brain homogenates were just as high as the α-synuclein intracellular levels [17]. These novel findings in combination with the previous reports suggesting that the gradual spread of synucleinopathy, described in the PD brain [4], might be due to cell-to-cell transmission of α-synuclein [18], [19] highly warrant investigations of the extracellular α-synuclein breakdown mechanisms. Several mechanisms of α-synuclein degradation, involving the ubiquitin-proteasome system and autophagy have been described but the major mechanism of α-synuclein degradation in neurons is still under debate [20]. Neurosin, a 244 amino acid protein, is one of many enzymes suggested to cleave α-synuclein. Initially, three research groups independently cloned the protein and named it zyme, neurosin and protease M [21], [22], [23] respectively. All three groups found the protein to be expressed in human brain tissue and additional studies confirmed that neurosin exists in high concentrations in various biological fluids such as human breast milk, nipple aspirate fluid and CSF [24]. The 244 amino acid pre-pro enzyme is proteolytically processed to an inactive pro-form by removal of a 16 amino acid signal peptide. Upon secretion the pro-enzyme is converted to an active protease by removal of a five amino acid signal peptide. Today there are several reports on the expression, regulation and physiology of neurosin as well as its diverse roles in pathobiology, thoroughly reviewed in the work by Bayani and Diamandis [25]. The α-synuclein degrading property of neurosin has convincingly been demonstrated in cell-free systems and in cultured cells [26], [27] and additional *in vitro* studies have shown that phosphorylated α-synuclein is more resistant to neurosin degradation than non-phosphorylated α-synuclein [28].

Neurosin has also been suggested to play a role in AD pathogenesis. The protein has been found in the pathological senile plaques of AD patients [29] and several different experimental studies have described its ability to cleave the amyloid precursor protein (APP) [22], [30], [31]. In human CSF, neurosin has been identified in its inactive proform [32] and examination of CSF, plasma and brain tissue extracts from AD patients versus controls have revealed altered neurosin expression and subsequently the protein was suggested as a potential AD biomarker [33], [34] as well as a risk factor for developing AD [35]. Few studies have thereafter in detail studied the potential mechanisms by which neurosin could be linked to the pathological processes occurring in AD and PD. In a recent publication by Spencer and colleagues it was demonstrated that decreased expression of neurosin was associated with increased accumulation of α-synuclein in postmortem brain tissue of PD/DLB patients as well as in α-synuclein transgenic mouse models [27]. The authors of that study further suggested that lentivirus vector driven expression of neurosin, which in their hands reduced α-synuclein pathology in a mouse model of Lewy body disease, should be further explored as a potential therapeutic tool for DLB. In light of the current focus on α-synuclein as a potential CSF biomarker of synucleinopathy the role of neurosin as a modulator of α-synuclein metabolism is again brought to the fore.

To our knowledge simultaneous quantification of α-synuclein and neurosin in CSF from patients with synucleinopathy versus non-demented controls and AD patients has not yet been performed. In the current study we therefore examined the levels of neurosin and α-synuclein in CSF from patients with AD, DLB, PD and PD with dementia (PDD) versus non-demented controls. We also evaluated potential links between the CSF levels of α-synuclein, neurosin and disease severity.

## Subjects and Methods

### Study Participants

Cerebrospinal fluid samples were obtained from non-demented controls (n = 52) and patients clinically diagnosed with AD (n = 46), PD (n = 38), PDD (n = 22) and DLB (n = 33) at the Neurology and Memory Clinics at Skåne University Hospital (Sweden). The patients diagnosed with PD met the NINDS Diagnostic Criteria for Parkinson Disease [36]. Patients diagnosed with PDD met the Clinical Diagnostic Criteria for Dementia Associated with Parkinson's Disease [37]. Patients who received an AD diagnosis (n = 46) had to meet the DSM-IIIR criteria of dementia (American Psychiatric Association 1987) and the criteria of probable AD defined by NINCDS-ADRDA [38]. Patients with DLB (n = 33) met the consensus criteria [39]. The controls did not fulfill the criteria of any neurological or dementia disorder. All individuals gave informed consent to participate in the study. The study was conducted according to the Helsinki Declaration and approved by the ethics committee of Lund University, Sweden.

#### Lumbar puncture

Lumbar puncture was performed in the L3/L4 or L4/L5 intervertebral space and CSF was collected passively by gravity flow in polypropylene tubes. Within 30 minutes the CSF was centrifuged at 4°C at 2000×g for 10 min. The supernatant was collected, gently mixed, aliquoted in polypropylene tubes and frozen within 2 h at −80°C pending biochemical analysis. The procedure followed the Alzheimer's Association Flow Chart for LP and CSF sample processing [40].

### Determination of CSF neurosin concentrations

#### Characterization of neurosin antibody

The specificity of the biotinylated polyclonal goat anti-human neurosin antibody (R&D Systems) against human neurosin (amino acids 22–244), was assessed by western blotting (WB). The same antibody was used to immunoprecipitate neurosin from pooled human CSF using a Protein G Immunoprecipitation Kit (Sigma Aldrich) according to the recommendations by the supplier. Neurosin was eluted in 0.1 M glycine buffer pH 2.5 and immediately neutralized with 1 M Tris pH 9.5. Recombinant human neurosin with a C-terminal 10 His-tag, expected to run at 35 kDa (R&D Systems), was separated along with the immunoprecipitated CSF neurosin by use of a 12.5% SDS-PAGE under reducing conditions. The separated proteins were transferred to a PVDF membrane (Millipore) by use of the trans-blot semi-dry transfer cell (Bio-Rad) where-after the membrane was blocked at 4°C over night (o/n) in phosphate buffered saline (PBS) with 0.5% skimmed milk (Fluka Analytical) and 0.1% Tween20 (Merck). For the WB analysis the membrane was probed with the goat anti-human neurosin antibody (diluted in PBS with 0.1% Tween20), rinsed and then further incubated with a donkey anti-goat IRDye secondary antibody (Li-Cor Biosciences). Immunolabeled proteins were visualized using the Odyssey Infrared Imaging System (Li-Cor Biosciences).

#### Neurosin ELISA

A direct neurosin enzyme-linked immunosorbent assay (ELISA) was used for the quantification of neurosin in CSF. Recombinant human neurosin (R&D Systems) (standard concentration range 1.25–80 ng/mL) and CSF samples were diluted in PBS and added in duplicate (100 µL) to the wells of a MaxiSorb 96-well polystyrene plate (Nunc A/S). Standards and samples were incubated o/n at 4°C after which they were discarded and the plate rinsed 3× with wash-buffer (PBS with 0.05% Tween20). After rinsing the wells were blocked with blocking buffer (PBS with 1% bovine serum albumin) for 1 h at room temperature (RT) followed by rinsing (see above) and incubation with detection antibody (biotinylated goat anti-human neurosin antibody, R&D Systems), diluted in blocking buffer, for 2 h at RT. After another rinse step HRP-streptavidin (R&D Systems) solution was added to the wells and the plate incubated for 20 min at RT. For the colorimetric reaction to occur peroxidase substrate (tetramethylbenzidine) (KBL) was added to the wells and after 20 min incubation in the dark the reaction was stopped with 1 M H_2_SO_4_. The optical density at 450/540 nm was determined using a microplate reader (Labsystems iEMS) with background correction. Readings of the standards and CSF samples were averaged and neurosin concentrations determined by use of a 4 parametric curve fit. The intra- and inter-assay variations coefficients (CV%) throughout the standard range were <10 and <15 and recovery of spiked standards (full standard curve range) into appropriately diluted CSF samples was between 95–110%. To limit the effect of inter-assay variations the results of the CSF samples were corrected against the results of repeated analyses of two control samples (pooled CSF).

### Quantification of CSF α-synuclein

Concentrations of α-synuclein were determined by use of a sandwich ELISA (Invitrogen) previously described [16]. In brief, neat CSF samples were assayed along with two control samples (pooled CSF). Recovery experiments were performed by spiking neat CSF with known concentrations of the provided standard. Recovery of the spiked α-synuclein concentrations was determined to range between 102–122%. The intra- and inter-assay CV% were determined by repeated analyses of the two undiluted control samples yielding CV% of 2.5 versus 7 respectively. To avoid inter-lot variation, assays of the same lot were used for the entire analyses.

### Statistics

For the statistical analyses the PASW Statistics 18 (SPSS Inc) software was used. The α-synuclein and neurosin data was normally distributed, assessed by the Kolmogorov-Smirnov test, therefore parametric tests were used throughout the analyses. The independent t-test was used to compare results between two groups (comparisons between gender and between apolipoprotein Eε4 allele (APOEε4) carriers and non-carriers) whereas the one-way ANOVA or ANCOVA, with correction for age, was used to compare results between more than two groups with adequate correction for multiple comparisons (Bonferroni). To analyze frequency differences of dichotomous variables the chi-square test was used. The Pearson correlation test was used to assess correlations between variables. Levels of neurosin, but not α-synuclein, were related to age and all analyses including neurosin were therefore adjusted for age. The data is presented as mean ± SD and p<0.05 was considered significant.

## Results

### Characteristics of study participants

The demographic data of the study participants is summarized in table 1. The non-demented controls and patients with PD were significantly younger than patients with AD, DLB and PDD (p<0.001). The gender distribution also varied between the diagnostic groups (p = 0.003) with the majority of the AD patients being female whereas the majority of the PDD patients were males. Furthermore, patients with mild to moderate dementia (AD, DLB and PDD) exhibited, as expected, significantly lower MMSE scores compared to controls (p<0.001) and PD patients (p<0.001). In order to simplify the discussion around patients with synucleinopathies table 1 also shows the demographic data of patients with DLB, PD and PDD grouped into one group of patients with synucleinopathy (SYN).

**Table 1:** Study Population Characteristics.

Characteristics	CON (n = 52)	AD (n = 46)	DLB (n = 33)	PD (n = 38)	PDD (n = 22)	SYN (n = 93)
Age (yrs)	64±10***	78±6	74±6	64±9***	75±6	70±9
Sex F/M (%)	50/50	76/24	55/45	47/53	27/73	45/55
Total MMSE	28.5±1.1	20.6±4.3	20.2±5.6	28.5±1.2	23.1±4.4	24.3±4.4
PD severity#	ND	ND	ND	2.3±0.8	ND	2.3±0.8†

*** p<0.001 when compared to AD, DLB and PDD.

# Modified Hoehn and Yahr Staging scale (0–5) [58].

† ) Only determined for the PD group. ND) not determined.

### Cerebrospinal fluid neurosin concentrations

**Figure 1:**
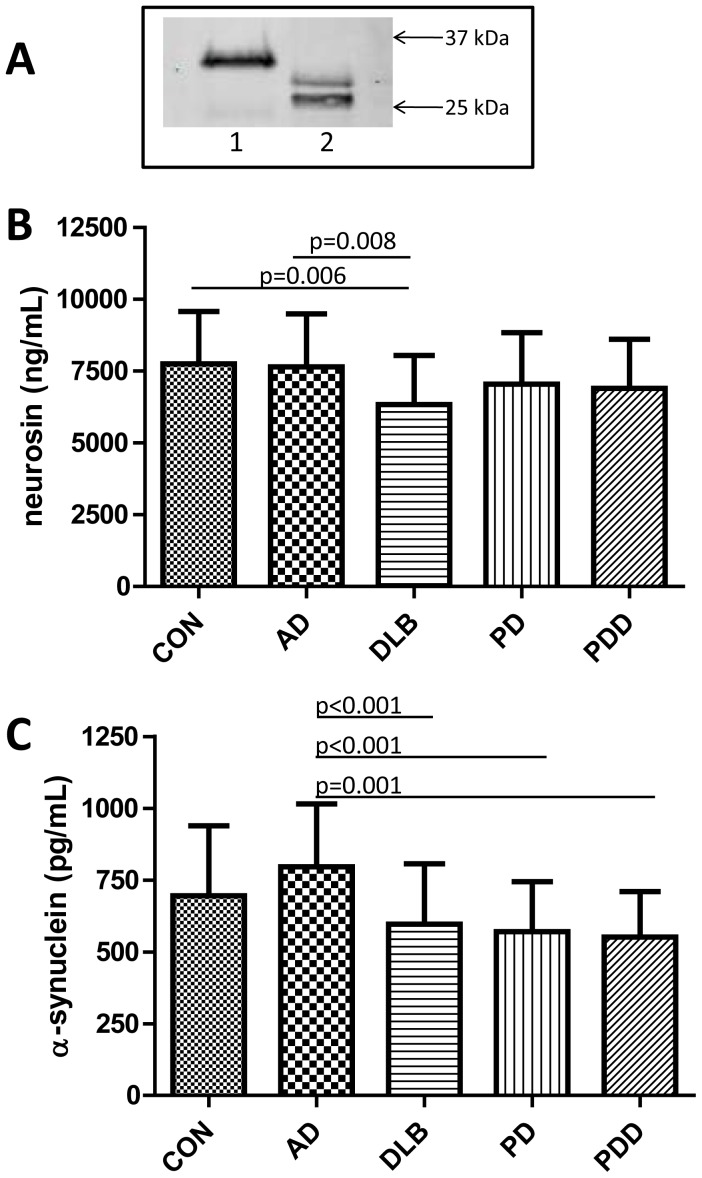
Specificity of the biotinylated goat anti-human neurosin antibody used in the neurosin ELISA was evaluated by western blotting (A). Lane 1 recombinant human neurosin, lane 2 immunoprecipitated neurosin from pooled human CSF. CSF concentrations of neurosin (B) and α-synuclein (C). Significant differences are indicated by p-values generated by use of ANCOVA group comparisons with correction for age or ANOVA followed by Bonferroni posthoc testing.

**Figure 2:**
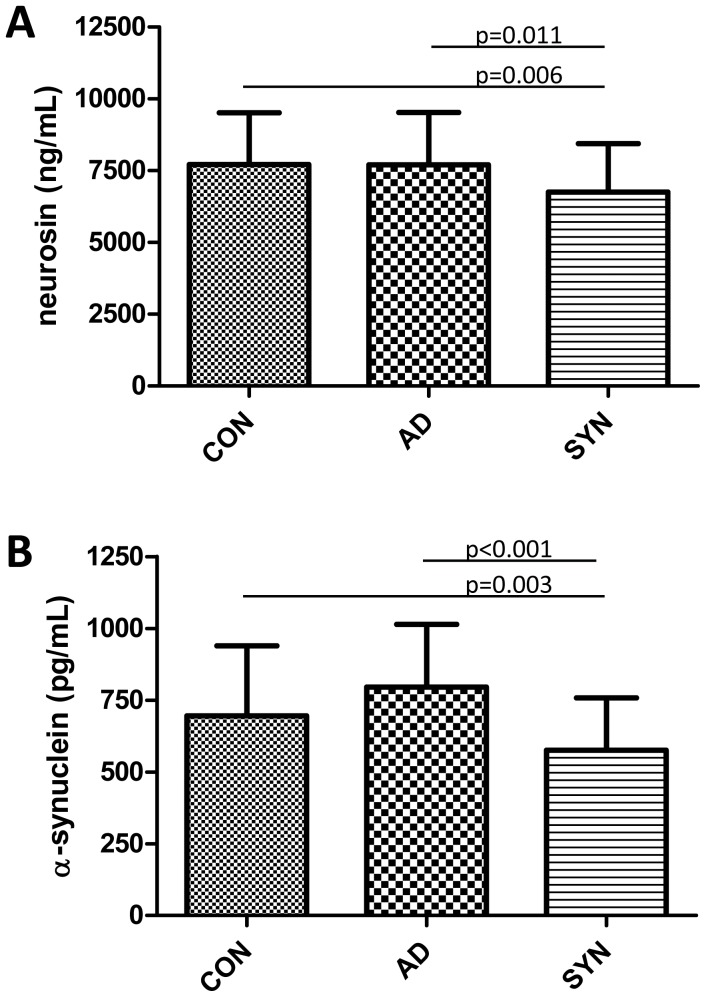
CSF levels of neurosin (A) and α-synuclein (B) in non-demented controls (CON), Alzheimer's disease patients (AD) and patients with Parkinson's disease, Parkinson's disease dementia and dementia with Lewy bodies grouped into one cumulative group of patients with synucleinopathy (SYN). Significant differences in CSF markers, as indicated by the p-values, were evaluated using ANCOVA group comparisons with correction for age or ANOVA followed by Bonferroni posthoc testing.

For the quantification of neurosin in CSF we developed and validated an in-house direct ELISA assay. The biotinylated goat anti-human neurosin antibody used in this assay detected the recombinant human neurosin (Glu17-Lys244 with a C-terminal 10 His Tag) at the expected migration position of ≈35 kDa (used as standard in our assay). The antibody also detected neurosin immunoprecipitated from pooled human CSF as a double band at approximately 26–30 kDa, as assessed by western blotting (figure 1A). Determination of neurosin in CSF showed that DLB patients exhibited the lowest levels (18% less than controls) amongst the investigated groups (figure 1B). Interestingly, neurosin levels in the DLB group were significantly less than in the controls and in the AD group (figure 1B). Neurosin concentrations were similar in the three groups with synucleinopathy (DLB, PD and PDD) and when pooled to one group of synucleinopathies (SYN) this group (n = 93) significantly differed from controls (figure 2 A). Since correlation analysis revealed that CSF neurosin was significantly linked to age in the control group (r = 0.317, p = 0.022) and the mean age of the investigated diagnostic groups was significantly different, the statistical analyses was corrected for age (results presented in figures 1 and 2 are age-adjusted). Presence of the APOEε4 allele did not affect neurosin levels and we did not observe any effect of gender in any of the investigated groups (data not shown).

### Cerebrospinal fluid α-synuclein concentrations

In order to determine the relationship between CSF neurosin and α-synuclein levels we determined the concentrations of the latter marker, by use of an assay previously employed in another CSF study [16]. Contrary to the CSF neurosin levels, concentrations of α-synuclein were not related to age. Amongst the diagnostic groups AD patients demonstrated the highest α-synuclein levels, 14% higher than the CSF α-synuclein determined in the control group (p = 0.034). This statistically significant difference was however lost when corrected for multiple comparisons (n = 10 group comparisons). The three groups of patients with synucleinopathy exhibited the lowest CSF α-synuclein (all less than 600 pg/mL) with the lowest levels found in patients with PDD and all were significantly lower than the α-synuclein levels found in the AD group (figure 1C) (AD>controls>DLB>PD>PDD). There was no difference in α-synuclein concentrations between the three synucleinopathies and when pooled (n = 93) this group (SYN) significantly differed from both controls and AD patients (figure 2B).

Our previously presented results suggested gender-specific variations in α-synuclein levels between AD and DLB patients versus controls [16]. In our present study, gender appeared linked to α-synuclein levels in patients with PD/PDD. In the PD group female patients demonstrated higher α-synuclein levels than males (634±181 versus 512±148 pg/mL, p = 0.029) whereas in the PDD group the results were inverse and higher in male versus female patients (593±164 versus 442±66 pg/mL, p = 0.043). Further, when comparing females and males, respectively, across the diagnostic groups female AD patients (n = 35, 785±200 pg/mL) significantly differed from female DLB (n = 18, 553±181 pg/mL, p = 0.004) and PDD patients (n = 6, 442±66 pg/mL, p = 0.005). When comparing male individuals across the groups, AD patients (n = 11, 830±280 pg/mL) differed from PD (n = 20, 512±148 pg/mL, p = 0.001) and PDD patients (n = 16, 593±164 pg/mL, p = 0.033) but not DLB patients (n = 15, 649±237 pg/mL, p = 0.253). We observed no influence of presence of the APOEε4 allele in any of the groups (data not shown).

### Variable associations

By use of correlations analysis we evaluated potential links between the CSF levels of both neurosin and α-synuclein, age, cognitive function (total MMSE score) and PD severity (modified Hoehn and Yahr staging scale). Earlier reports have described a link between age and CSF levels of neurosin [35]. We found a positive association between age and neurosin levels in non-demented controls and in AD patients (table 2) but not in patients with synucleinopathies. Also CSF levels of α-synuclein have earlier been associated with age [10]. In our recent study on patients from the Malmö Alzheimer Study [16] we did not find any evidence of such a link and in the present study we found a positive association between age and α-synuclein only in the PD group (table 2). We found no indication of any links between α-synuclein, neurosin and PD severity (data not shown). Interestingly, CSF neurosin and α-synuclein were correlated in non-demented controls and positive correlations were also found in PD and PDD patients but not in patients with AD or DLB (table 2).

**Table 2:** Neurosin and α-synuclein associations.

	Age	MMSE	CSF α-synuclein
**CSF Neurosin**	**r**	**r**	**r**
CON	0.317*	---	0.527***
AD	0.371*	---	---
DLB	---	---	---
PD	---	---	0.395*
PDD	---	---	0.421*
**CSF α-synuclein**	**r**	**r**	
CON	**---**	**---**	
AD	**---**	**---**	
DLB	**---**	**---**	
PD	0.356*	**---**	
PDD	---	**---**	

Correlation (r) significant at the level of

* p = 0.05,

** p = 0.01 and

*** p = 0.001,

--- no significant correlation.

In order to evaluate potential associations between neurosin, α-synuclein and the established AD biomarkers, as additional indicators of disease severity, we compiled AD biomarker data from a subset of the individuals included in our study and of whom the CSF had undergone routine analysis for Aβ42, t-tau and p-tau. Few of the PD and PDD patients had undergone routine screening for CSF AD biomarkers and these individuals were pooled into one group (PD/PDD n = 11 since PD n = 2 and PDD n = 9). The AD biomarker data is presented in table 3. As expected, patients with AD displayed the lowest Aβ42 levels (table 3). However, also the DLB and PD/PDD group exhibited significantly lower Aβ42 levels compared to controls. Patients with AD demonstrated significantly higher t-tau and p-tau levels compared to all other groups of which CSF tau levels were unaltered compared to controls (table 3).

**Table 3:** CSF levels of AD biomarkers.

AD biomarkers	CON (n = 33)	AD (n = 35)	DLB (n = 30)	PD/PDD (n = 11)
CSF Aβ1–42 (ng/L)	669±237	393±79***	448±191***	458±204**
CSF t-tau (ng/L)	273±138	835±339#	405±174	335±127
CSF p-tau (ng/L)	47±19	102±38#	58±22	54±40

*** p<0.001 compared to controls,

** p = 0.007 compared to controls,

# p<0.001 compared to controls, DLB and PD/PDD.

Next we analyzed potential links between neurosin, α-synuclein and CSF AD biomarkers in controls, AD, DLB and the PD/PDD group. Results from the correlation analyses are shown in table 4. In controls both neurosin and α-synuclein were significantly associated with all three AD biomarkers (table 4), further highlighting the link between these two markers (table 3). Also, neurosin was linked to levels of p-tau in all groups with the strongest associations found in controls and PD/PDD patients (table 3). Neurosin levels were not linked to t-tau levels in any of the investigated patient groups. In contrary, patients with AD and DLB but not PD/PDD exhibited significant associations between t-tau and α-synuclein. In line with the results of neurosin also α-synuclein levels were positively associated with levels of p-tau in all groups (table 3).

**Table 4:** Neurosin and α-synuclein associations with AD biomarkers.

	CSF Aβ1–42	CSF t-tau	CSF p-tau
**CSF Neurosin**	**r**	**r**	**r**
CON	0.495**	0.388*	0.545***
AD	---	---	0.375*
DLB	---	---	0.378*
PD/PDD	---	---	0.752*
**CSF α-synuclein**	**r**	**r**	**r**
CON	0.514**	0.767***	0.895***
AD	---	0.780***	0.676***
DLB	---	0.573***	0.567***
PD/PDD	---	---	0.841***

Correlation (r) significant at the level of

* p = 0.05,

** p = 0.01 and

*** p = 0.001,

--- no significant correlation.

## Discussion

In the current study we elucidated whether CSF levels of neurosin and its substrate α-synuclein differ between patients with synucleinopathy, DLB, PD, PDD, and patients with AD, versus non-demented controls. Our results revealed that all three disease groups with synucleinopathy exhibited similar neurosin levels. When pooled, the CSF neurosin concentrations in the combined group of total synucleinopathies were significantly lower compared to neurosin found in both AD patients and non-demented controls. Interestingly, the lowest neurosin concentrations were found in the DLB group, which differed significantly from AD patients and controls. In agreement with recent studies we also show 14% higher levels of α-synuclein in AD patients versus controls however in the absence of altered neurosin levels.

To our knowledge we are first to describe decreased neurosin levels in CSF from patients with synucleinopathy disorders versus controls and AD patients. In support, we have found similar results in a previous study on CSF samples from a different cohort (randomly selected sample from the Malmö Alzheimer Study) [41]. That study included patients with DLB (n = 26), AD (n = 26) and age-matched elderly controls (n = 26) and showed reduced CSF neurosin levels in DLB patients compared to AD patients but not controls [41]. Nevertheless, follow-up studies in larger, preferably age-matched, patient cohorts are strongly needed to confirm our results. Previous immunohistological studies have shown decreased neurosin immunoreactivity in neurons as well as colocalization of Lewy bodies and neurosin immunoreactivity in the PD brain [29]. In support, Spencer and colleagues reported a more than 50% reduction in neurosin expression in the temporal cortex from DLB patients versus non-demented controls. In line with our results the same authors described neurosin as a double band of approximately 26 kDa when analyzing brain tissue homogenates. Results from the same study also showed that lentivirus driven expression of neurosin could promote α-synuclein clearance and reduce pathology in α-synuclein transgenic mouse models [27]. Together these results further strengthen the proposed link between α-synuclein pathology and neurosin.

Neurosin levels in CSF from AD patients and controls have previously been investigated in two different studies. These two studies employed different ELISA assays [33], [35] and presented conflicting results. The first study showed three fold higher CSF neurosin concentrations in AD versus controls [33] whereas the second study, similarly to our current study, showed no difference in neurosin levels in AD patients compared to controls [35]. Low numbers of investigated patients (low statistical power) as well as disease severity could have influenced the different study outcomes. The lack of consistency could possibly also be explained by the fact that the two studies used different quantification assays and therefore may have identified different isoforms of neurosin, which recently was shown to exist as three splice variants [42]. The significance of these three variants remains to date elusive. Further, different immunoassays may also detect the three different activity forms (the pre-pro and pro-forms or the mature active protease) with varying affinity. The antibody employed in the current study most likely detects two forms as indicated by the double band detected at approximately 26–30 kDa. Only one study has so far aimed to determine the form of neurosin in the CSF. That particular study by Okui and colleagues suggested that neurosin is present in the inactive pro-form in the CSF [32]. Further studies however would need to confirm those results as our study suggests the presence of at least two neurosin forms in CSF. Future studies should also address the question of neurosin activity and the balance of inactive versus active neurosin pools in relation to accumulation of α-synuclein in the brain. Several studies have demonstrated consistent findings in terms of neurosin expression, immunoreactivity and concentrations in AD brain tissue. For instance, the relative neurosin mRNA levels in AD brains was shown to be lower than in controls [29] and brain tissue extracts from AD patients contained approximately two-fold lower neurosin protein concentrations than brain tissue extracts from controls [33]. A recent study additionally showed that neurosin expression and protein concentrations were decreased in the frontal but not temporal cortex in AD patients versus controls [43]. Thus, neurosin concentrations might vary between brain regions and we speculate that altered neurosin levels in specific brain regions may be undetectable in CSF as these changes might be masked by the total amount of neurosin in the brain. This hypothesis would also suggest that changes in neurosin levels only are detectable after major changes in neurosin expression potentially occurring in the late stages of AD.

In support of the earlier proposed link between AD and neurosin, we here described a correlation between neurosin and Aβ42 levels in CSF from controls. This association supports a role for neurosin in the Aβ pathway, at least in non-demented controls, which however might be rendered upon disease, as earlier suggested by experimental data [22]. Controls also showed correlations between neurosin and the AD markers t-tau and p-tau. In addition, correlations between neurosin and p-tau were found in all patient groups whereas correlations between neurosin and t-tau were absent. What role neurosin might play in the pathway of Aβ and phosphorylated tau is yet to be elucidated, but immunohistochemical studies demonstrated that neurosin colocalized with plaques and tangles in the AD brain, which could indicate that neurosin secreted from surrounding cells attach to the pathological structures for proteolysis [29].

The number of studies reporting CSF concentrations of α-synuclein in patients with synucleinopathy is rapidly increasing. Whereas few studies have reported unaltered levels of α-synuclein in these patients [44], [45] most studies have reported decreased CSF α-synuclein concentrations in patients with either DLB or PD versus controls, but also versus AD (for review see [1]). We found the highest α-synuclein levels in AD patients whereas patients with DLB, PD or PDD all exhibited significantly lower α-synuclein. The same pattern of increased α-synuclein in AD and decreased levels in patients with PD, DLB and MSA was recently demonstrated by Tateno and colleagues [14]. Importantly, Larson and colleagues recently showed that brain tissue from AD patients, in the absence of Lewy body pathology, contained almost two-fold higher soluble α-synuclein concentrations compared to control brains. In the same study the authors showed that soluble α-synuclein levels were more strongly associated with cognitive impairment than soluble levels of tau and Aβ. These recent data position α-synuclein as a potential disease player also in AD pathophysiology [17].

The results presented in the current study are the first to demonstrate that decreased CSF neurosin concentrations are significantly associated with decreased α-synuclein concentrations in the CSF of controls and patients with PD and PDD but not AD and DLB. These findings are to date difficult to interpret since little is known about the relationship between the extracellular and intracellular pool of neurosin and α-synuclein as well as the association between the brain tissue concentrations and quantities of these proteins in the CSF. As suggested in the recent report by Larson and colleagues the pool of soluble α-synuclein in the brain tissue of AD patients may in fact be significantly increased [17]. With increased levels of α-synuclein also in the CSF of AD patients, as shown in our present study, an increase of this protein may in fact mirror elevated levels of α-synuclein in the brain tissue. Biomarker studies in the AD field, using positron emission tomography (PET) and Aβ-binding tracers like the Pittsburgh Compound B (PIB) have demonstrated that decreased CSF concentrations of Aβ42 *in vivo* mirror increased amyloid deposition in the brain [46]. Whether decreased CSF α-synuclein levels indeed reflect increased deposition of α-synuclein in the brain remains to be investigated. In support of the results demonstrated by Spencer and colleagues [27] we speculate that altered neurosin production or activity might hamper α-synuclein clearance, possibly of specific α-synuclein aggregation forms, contributing to α-synuclein Lewy pathology seen in synucleinopathy patients. Interestingly, our correlation analysis further showed that neurosin and α-synuclein levels were unrelated in AD and DLB patients even though the latter group, just like PD and PDD patients, displayed lower levels of both neurosin and α-synuclein. Careful consideration of the data would suggest that potential co-existing AD pathology in DLB patients who frequently share neuropathological characteristics of both PD and AD [47], may mask potential correlations between neurosin and α-synuclein in the CSF.

Correlations between α-synuclein and the AD biomarkers Aβ42, t-tau and p-tau in synucleinopathy patients have lately been investigated and the combination of CSF α-synuclein, tau and Aβ1–42 have shown promising results for facilitated discrimination of synucleinopathy [13], [48], [49], [50]. In our study we found a strong positive correlation between tau and α-synuclein in all investigated groups. These results are in line with results previously published by us and others, but contradict one study describing negative correlations between the two proteins [16], [49], [51]. Inclusion of patients with different disease severity and the use of different α-synuclein quantification methods might result in these discrepancies. The significance of the repeatedly reported link between tau and α-synuclein has yet to be investigated, but novel studies have proposed that α-synuclein promotes intracellular aggregation and phosphorylation of tau [52], [53], [54]. Further, in the study by Larson and colleagues the authors proposed that synergism between Aβ/APP and human tau may underlie abnormal elevation of soluble α-synuclein in transgenic mice. Elevated levels of soluble α-synuclein in that study led to alterations of the protein composition of synaptic vesicles impairing neurotransmitter release [17]. These observations are in line with several studies proposing important implications of α-synuclein in synaptic plasticity, recycling of synaptic vesicles and neurotransmitter synthesis, storage and release (recently reviewed in [55]). Results from other experimental studies also showed that tau is secreted through an exosome-mediated release mechanism [56], similar to α-synuclein [57]. It is therefore interesting that α-synuclein has been shown to co-precipitate with tau, which further suggests that tau and α-synuclein in solution might exist as a complex [53]. The described link between tau and α-synuclein has further lead to the suggestion that CSF α-synuclein, similar to CSF levels of tau, could function as a marker of synapse loss and neurodegeneration [51].

We recently demonstrated significantly lower α-synuclein in female, but not male patients with DLB versus AD patients and controls [16]. In the current study we observed similar results, i.e. female but not male DLB patients differing significantly from AD patients. We also demonstrated variations within the PD and PDD groups, where male PD but female PDD patients exhibited the lowest α-synuclein levels compared to patients of the opposite gender. Despite the strong correlations between CSF levels of neurosin and α-synuclein in controls and patients with PD and PDD we observed no gender-effect on neurosin concentrations. The significance of these observed gender-differences is yet to be determined in larger cohort-studies.

To summarize, data from recent reports in combination with our current findings of lower versus elevated CSF levels of α-synuclein in patients with synucleinopathy versus AD respectively and decreased levels of neurosin in the former but not the latter group, may point to different disease-specific neuropathological mechanisms in these disease groups. Alterations in neurosin production or activity might hamper clearance of specific α-synuclein forms leading to Lewy pathology in patients with synucleinopathy but not AD. Last, we propose neurosin as a potential marker of synucleinopathy and a natural candidate in studies elucidating whether a specific panel of CSF proteins will aid the identification, discrimination and differential diagnosis of various synucleinopathies versus AD.
